# A Bayesian approach to efficient differential allocation for resampling-based significance testing

**DOI:** 10.1186/1471-2105-10-198

**Published:** 2009-06-28

**Authors:** Shane T Jensen, Sameer Soi, Li-San Wang

**Affiliations:** 1 Department of Statistics, The Wharton School, University of Pennsylvania, Philadelphia, PA, 19104 USA; 2 Genomics and Computational Biology Program, University of Pennsylvania, Philadelphia, PA, 19104 USA; 3 Penn Center for Bioinformatics, University of Pennsylvania, Philadelphia, PA, 19104 USA; 4 Department of Pathology and Laboratory Medicine, University of Pennsylvania, Philadelphia, PA, 19104 USA

## Abstract

**Background:**

Large-scale statistical analyses have become hallmarks of post-genomic era biological research due to advances in high-throughput assays and the integration of large biological databases. One accompanying issue is the simultaneous estimation of p-values for a large number of hypothesis tests. In many applications, a parametric assumption in the null distribution such as normality may be unreasonable, and resampling-based p-values are the preferred procedure for establishing statistical significance. Using resampling-based procedures for multiple testing is computationally intensive and typically requires large numbers of resamples.

**Results:**

We present a new approach to more efficiently assign resamples (such as bootstrap samples or permutations) within a nonparametric multiple testing framework. We formulated a Bayesian-inspired approach to this problem, and devised an algorithm that adapts the assignment of resamples iteratively with negligible space and running time overhead. In two experimental studies, a breast cancer microarray dataset and a genome wide association study dataset for Parkinson's disease, we demonstrated that our differential allocation procedure is substantially more accurate compared to the traditional uniform resample allocation.

**Conclusion:**

Our experiments demonstrate that using a more sophisticated allocation strategy can improve our inference for hypothesis testing without a drastic increase in the amount of computation on randomized data. Moreover, we gain more improvement in efficiency when the number of tests is large. R code for our algorithm and the shortcut method are available at .

## Background

### Nonparametric tests in multiple hypothesis testing scenarios

Large-scale statistical analyses have become hallmarks of post-genomic era biological research due to advances in high-throughput assays and the integration of large biological databases. As the analysis becomes larger and more complex, various kinds of computational issues arise. The context of our investigation is *multiple testing*, the simultaneous estimation of p-values for a large number of hypothesis tests. For example, in a typical control-treatment microarray experiment, the goal of the analysis may be to identify target genes by applying the same testing procedure on each of the genes and selecting those that show the most extreme differential expression.

Most multiple testing scenarios involve the assumption of a parametric null distribution (such as the normal or *t *distribution) for each observed test statistic. However, in many applications, this parametric assumption may be unreasonable, resampling-based p-values are the preferred procedure for establishing statistical significance. For example, in the usual permutation test framework, resamples are generated by randomly permuting the treatment and control labels among the available data samples. We then calculate the test statistic for each of these resamples and calculate the p-value for each gene as the fraction of the resamples that have more extreme test statistics than the observed test statistic for that gene. Ideally, we would be able to evaluate the test statistic for every possible resample, and thus calculate the resample-based p-value exactly. However, this is usually not feasible for datasets involving many replicates, so the usual procedure is to use Monte Carlo simulation to estimate each p-value based on a large set of resamples. As an example, an option in the popular SAM microarray analysis software [1] allows the user to use permutation tests to assess the p-value without the normality assumption. A similar re-sampling scheme for estimating p-values can be based on the bootstrap. In [2], a nonparametric test procedure is applied to every gene to examine how well the expression profile of a gene (say over a time course) fits some preset order-restrictions; the p-value of the test is obtained using 50000 bootstrap resamples *per gene*. We refer the reader to [3,4] for the rationale and more details on bootstrapping, permutation tests, and other nonparametric tests. In this paper we collectively refer these methods as *resampling *procedures and a randomly generated sample (whether bootstrap or permutation-based) is called a *resample*.

This paper focusses on the following setting: we have *N *units (eg. genes), and we want to conduct a hypothesis test for each gene *i *based on observed test statistic *T*_*i*_. We do not want to make any parametric assumptions about this test statistic, so the p-value *p*_*i *_for each test needs to be estimated by a resampling procedure. The additional element that is implicit in our framework is that the number of tests *N *is large (can be as high as 10^6 ^for genome-wide association studies), so we need to control for the large number of tests being performed. Many multiple testing procedures focussed on control of the *family-wise error rate *(FWER), with a popular choice being the Bonferroni correction [5]. More recently, the focus in multiple testing procedures has shifted to control of the *false discovery rate *(FDR) [6-8], which is much less conservative than FWER-control procedures. Since this current work was motivated by biological applications, we will use the terms *gene *and *unit *interchangebly, with the understanding that our methods are applicable to any multiple testing situation.

### Typical Uniform Resampling Strategies

Typical resampling procedures for p-value estimation use an equal number of resamples, say *B*, assigned to each of *N *genes, for a total of *N *× *B *resamples. Even in the simple framework where each gene will be assigned the same number of resamples, there are several alternative strategies for resampling-based inference. The first issue is whether each resample should be performed by randomly permuting the treatment and control labels of an entire column (across all genes) of data values, with the alternative being that treatment and control labels are permuted within each gene independently. We refer to the first strategy as a *column-wise *procedure and the second strategy as an *gene-independent *procedure. Many recent investigations (eg. [9-11]) argue for column-wise resampling procedures in order to retain potential dependencies between genes. Other recent microarray investigations (eg. [12]) have employed gene-independent resampling procedures. Clearly, a column-wise resampling procedure allocates resamples to all genes simultaneously, which implies a uniform allocation of resamples across genes. Although this column-wise strategy is preferred in certain situations, it suffers from the same inefficiencies as any uniform allocation procedure: genes that are clearly distant from the decision threshold will receive the same number of resamples as genes that are quite near the threshold. We focus on a gene-independent resampling procedure since it provides a more flexible framework for differential allocation of resamples among genes, which is the primary motivation for our current work.

Another issue is whether or not to combine resampled test statistics across genes when estimating the p-value for each gene. Many researchers (eg. [7]) prefer a *concatenation *procedure that uses all available resampled test statistics (across all genes) to achieve a higher resolution on the resampling-based null distribution when estimating each p-value. Since all resampled test statistics (across all genes) are used for each p-value calculation, there is little distinction between resampling strategies based on uniform allocation of resamples across genes versus differential allocation of resamples across genes. However, a concatenation procedure is only reasonable when the resampling-based null distribution is similar across genes, which is an uncomfortable assumption in many applications, such as genome-wide association studies when the allelic frequencies vary across loci. In these applications, a non-concatenation or *gene-separate *procedure would be preferred. Recent work (eg. [13,14]) proposes concatenation of statistics across only subsets of genes to correct for the fact that the null distribution is likely to differ between significant and non-significant genes. In this paper, we will focus on situations where *gene-separate *(non-concatenation) procedures are preferred, which is the area where differential allocation of resamples provides a substantial efficiency gain over a uniform allocation strategy.

In most multiple testing situations the vast majority of units are truly non-significant, which means that a uniform allocation strategy is devoting a large proportion of resamples to test statistics that are not even close to significant. For most estimated p-values that are quite large or extremely small (ie. far away from our decision threshold *p*_0_), then we are reasonably confident about our decision based on those p-values without need for a high degree of p-value accuracy (large number of resamples). Instead, we should focus a larger number of resamples on the estimation of p-values that are near to our decision-making threshold. For example, one may limit the number of resamples for a gene when the number of resamples with test statistic exceeding the actual statistic is larger than *p*_0 _× *B*, since the p-value of this gene will definitely be higher than the threshold *p*_0 _when all *B *resamples are computed. The gene is clearly nonsignificant, so we can stop evaluating more resamples for this gene and save computational time. This simple heuristic, which we call the *shortcut *approach, has been discussed previously [15] and implemented more recently in the popular software PLINK [16].

In this paper, we develop a principled iterative procedure for allocating different numbers of resamples to each unit. The overall intuition behind our approach is similar to the shortcut method in that we want to preferentially allocate more resamples to genes which have "borderline" p-values, i.e., p-values near to our classification threshold. The main difference is how the resample allocations are determined: we use a Bayesian-inspired approach that assigns resamples to each unit based on its individual "risk", the chance that the current p-value estimate leads to a misclassification of the unit. The goal is to lower the numbers of classification errors, since we are giving a higher resolution to the null distribution of genes that are more likely to be misclassified in a uniform allocation setting. This higher resolution comes at the sacrifice of resamples to non-borderline genes that should not need a very resolute null distribution for correct inference.

A detailed description of our differential allocation procedure is provided in the Methods section. The Results Section includes an experimental comparison that demonstrates the gains of our procedure over uniform procedures using two publicly available datasets: one microarray dataset on breast cancer [17], and one genome-wide association study [18] where computational efficiency in p-value estimation is a necessary concern due to its size. Our procedure maintains a low error-rate (low rates of false positives and false negatives) while using substantially fewer resamples in total. We then provide an additional experimental comparison to demonstrate that our method outperforms the shortcut method.

## Methods

### Differential Allocation of Resamples Using Risks

We separate the description of our procedure into several subsections for clarity of presentation.

#### Algorithm Initialization

The input data for our algorithm is an *N *× *J *matrix of data values, where *N *is the number of genes and *J *is the number of observations per gene. Our algorithm is initialized by a uniform allocation *burn-in *round, in which we assign *B*_0 _resamples to each gene, where *B*_0 _is a proportion of the *B *resamples that would be assigned to each gene by the typical uniform resampling procedure. Each of these resamples gives us a test statistic under the resampling-based null distribution, which we can use to get an initial p-value estimate for each gene.

Based on the given threshold *p*_0 _and our current estimated p-values , we have the current classification for each gene *i*: gene *i *is significant if  ≤ *p*_0 _or gene *i *is non-significant if  > *p*_0_. In case when *p*_0 _is determined using other criterion such as FDR, we use these p-value estimates to calculate our decision threshold *p*_0 _using the original FDR-control procedure proposed by [19].

#### Differential Allocation

**Figure 1:**
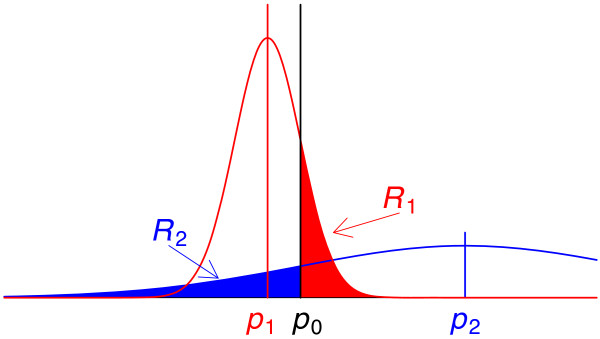
**Sample figure title**. Illustration of the risks associated with two different p-values. The red density is the posterior distribution of p-value *p*_1_. The blue density is the posterior distribution of p-value *p*_2_. The decision threshold for assessing significance is denoted as *p*_0_. The risk *R*_1 _(associated with p-value *p*_1_) is the red area on the right of the decision threshold *p*_0_. The risk *R*_2 _(associated with p-value *p*_2_) is the blue area on the left of the decision threshold *p*_0_.

Our algorithm now proceeds sequentially through multiple rounds and in each round a total of *K *new resamples are assigned. We want to allocate new resamples differentially to each gene *i *with the goal of minimizing the expected number of *mis-classified *genes ie. either non-significant genes that are inferred to be significant (false positives) or significant genes that are inferred to be non-significant (false negatives). Our framework treats either type of error (false-positives vs. false-negatives) as equally bad, though our approach could be easily generalized to differentially weight the two types of errors. Our proposed strategy is to assign new resamples with probability proportional to the *risk R*_*i *_of each gene *i*: the current probability of that gene *i *being misclassified.



where *p*_*i *_represents the true p-value for gene *i*. Only one of these two terms is non-zero, since any gene *i *can only be considered as either a false positive or false negative (not both) based on its current estimated p-value . We estimate the probabilities P(*p*_*i *_≤ *p*_0_) and P(*p*_*i *_> *p*_0_) based on the posterior distribution of p-value *p*_*i *_for gene *i*. Let *n*_*i *_be the number of resamples already performed on gene *i *and let *a*_*i *_be the number of resample test statistics that are more extreme than the observed test statistic for gene *i*. This pair of numbers (*a*_*i*_, *n*_*i*_) contains all the information we currently have for gene *i*. Assuming a uniform prior on each *p*_*i*_, *p*_*i *_~ Beta(1, 1), and with a binomial likelihood for our extreme resample counts *a*_*i *_~ Bin(*n*_*i*_, *p*_*i*_), then we have:

(1)

so each probability *R*_*i *_becomes

(2)

where B(*x*, *a*, *b*) is the CDF of the Beta(*a*, *b*) distribution evaluated at *x*. B(*x*, *a*, *b*) is often also referred to as the *incomplete Beta function*. In Figure 1, we see the risk for different locations of the posterior distribution *p*(*p*_*i*_|*a*_*i*_, *n*_*i*_). This illustration shows that the risk *R*_*i *_is the amount by which the posterior distribution (1) for gene *i *overlaps the significance threshold *p*_0_.

After the end of each round, *K *new resamples have been assigned proportional to the risks given in (2), and for each affected gene, the new p-value *p*_*i *_and the risk *R*_*i *_must be calculated. The algorithm stops when the total number of resamples assigned reaches a preset cap *B*_*tot*_. We should note that the above scheme considers the decision threshold *p*_0 _to be fixed and known, when it is actually itself an estimated quantity. A more general procedure that acknowledges the uncertainty in both the p-values *p*_*i *_and decision threshold *p*_0 _for FDR is the focus of continuing research. We provide a more detailed description of our proposed differential allocation algorithm below.

**Input**: Microarray measurements ***g***_*i *_= (*g*_*i*1_, ... *g*_*iJ*_) for each gene *i*, 1 ≤ *i *≤ *N*.

**Output**: Set of significant genes as defined by threshold *p*_0_.

#### Parameters

1. *B*_0_: number of reseamples per gene for burn-in.

2. *B*: average number of resamples to allocate per gene, so that *N *× *B *is total number of resamples to be used.

3. *K*: number of resamples allocated in each round.

#### Algorithm

1. For each gene *i*, compute observed test statistic *f*_*i *_= *f *(***g***_*i*_).

2. *Burn-in Allocation*: *n*_*i *_← *B*_0_

3. *Iterative Allocation*: Repeat:

(a) For each gene *i*, calculate *a*_*i *_= number of *n*_*i *_resamples with test statistic ≥ *f*_*i *_and set  = *a*_*i*_/*n*_*i*_

(b) For each gene *i*, compute *R*_*i *_← B(*p*_0_, *a*_*i *_+ 1, *n*_*i *_- *a*_*i *_+ 1). If *p*_*i *_≥ *p*_0 _then set *R*_*i *_← 1 - *R*_*i*_.

(c) For each gene *i*, compute *w*_*i *_= *R*_*i*_/Σ_*i*_*R*_*i*_.

(d) While *j *<*K*:

i. Select a gene *b *from the set (1, 2, ... *N*) with probability (*w*_1_, *w*_2_, ..., *w*_*N*_)

ii. Assign a resample to selected gene *b*: *n*_*b *_← *n*_*b *_+ 1

iii. *j *← *j *+ 1

4. Output the set of significant genes by applying threshold *p*_0 _on final set of {}.

### The Shortcut Method

An alternative differential allocation idea that we call the *the shortcut method *is to stop allocating resamples to any genes which have already accumulated enough non-extreme test statistics to guarantee that the null hypothesis for those genes will not be rejected. [15] discuss a sequential shortcut method for Monte Carlo estimation of p-values and more recently a shortcut method has been implemented in [20]. The popular software PLINK [16] for genome-wide association studies allows for more sophisticated approaches, such as using a confidence interval of the estimated p-value of a unit to decide if more resamples are needed.

Again let *N *be the number of genes and let *B *be the number of resamples that we would allocate to each gene in a uniform allocation scheme, so that we have a total of *N *× *B *resamples available to us. We again consider an iterative scheme where *n*_*i *_is the number of resamples already performed for gene *i *and let *a*_*i *_is the number of resample test statistics that are more extreme than the observed test statistic for gene *i*. If a particular gene *i *has accumulated enough non-extreme resample test statistics, i.e. if (*n*_*i *_- *a*_*i*_) > *B*·*p*_0_, then the resampling-based p-value  is guaranteed to exceed the threshold *p*_0 _and so allocating any more resamples to gene *i *is pointless. All remaining (*B *- *n*_*i*_) resamples that we would have devoted to gene *i *can now be allocated to other genes that still have a chance of rejecting the null hypothesis. This shortcut approach clearly differs from our proposed method in terms of how resamples are differentially allocated, but both should still be more efficient than a uniform allocation scheme. Another major difference is that our differential allocation method will also assign fewer resamples to genes when the p-value is much lower than the cutoff, whereas the shortcut method always tends to allocate more resamples to genes with a lower p-value.

### Experimental Comparision

#### Application to a breast cancer microarray dataset

The Hedenfalk et al. breast cancer dataset [17] consisted of 7 sporadic cases, 7 cases with BRCA1 mutations, and 8 cases with BRCA2 mutations. Following the guidelines in [7], we only examine samples associated with either BRCA1 and BRCA2 mutations, which results in 8 samples for BRCA1 and 7 samples for BRCA2. Following the preprocessing procedure in [7], we log_2_-transformed all measurements and removed outlier genes (defined as genes having any expression level above 20 in [7]); this left us with 3170 genes for further analysis. The subjects were divided into two groups. For each gene, we tested whether the mean expression levels of the two groups are significantly different. We used the absolute value of the Student's t-statistic and used permutation tests to compute the significance: the p-value of the gene is the fraction of random permutation resamples with larger statistic scores than the correct grouping of subjects. We varied the number of resamples per gene to see how the p-value estimation of our algorithm and the uniform allocation improved as the number of resamples increased. We assessed the accuracy by computing, as a reference, the exact p-values calculated by enumerating all  = 6435 possible resamples for each gene. The error of any p-value estimation is the number of genes mislabeled as significant or nonsignificant when compared with the significance calls using these reference exact p-values and a significance threshold of 0.0001. All computations were done using the R statistical software [21].

### Application to a Parkinson disease genome-wide association study dataset

The Parkinson's dataset [18] consisted of the genotype information of 402,582 SNPs on 271 cases and 270 controls. We randomly partitioned the dataset into 30 subsets of 13,626 SNPs each on average, and applied our algorithm to each subset separately. For each SNP, we used the chi-square statistic for the 3 × 2 contingency table, and computed the exact chi-square test p-value with 2 degrees of freedom as the "reference" p-value. We also applied our differential allocation algorithm by setting *B *= 1000, *B*_0 _= 100, 250, 500, 1000 (uniform allocation), and *p*_0 _= 10^-4 ^in the differential allocation algorithm. We then computed the accuracy and false discovery rate of the output from the four allocation algorithms using different p-value cutoffs; the "reference" set of significant SNPs were determined using the "reference" p-value using the same p-value cutoff.

#### Simulation study to compare our algorithm and the shortcut method

We compared our method and the shortcut method using the following parameter settings:

1. We use *N *= 300, 000, typical for genome-wide association studies. The actual p-values of all markers are generated as follows. First, for each marker we randomly sample an integer between 1 and *N*; the p-value of the marker is this number divided by *N*. Thus each marker will have a p-value between 1/*N *and 1 at this moment. We then replace the p-values of five of the markers by 10^-7 ^to represent real significant markers.

2. For both methods, we use the same p-value cutoff settings:

10^-5^, 2 × 10^-5^, 5 × 10^-5^, 10^-4^, 2 × 10^-4^, 5 × 10^-4^, 10^-3^.

3. For the shortcut method, each iteration allocates *B *= 10 resamples. The algorithm stops when the average number of resamples per marker exceed 100.

4. We use a simplified version of the adaptive permutation algorithm in PLINK, a program widely used in the analysis of genome-wide association studies [16]. At each iteration, the p-value estimate of marker *i *is  = (1 + *D*_*i*_)/(2 + *F*_*i*_), where *F*_*i *_is the total resamples allocated to *i *so far, and *D*_*i *_is the number of such resamples that yield higher statistics than the actual statistic (this is determined in the simulation by Bernoulli trials with success probability *p*_*i*_). This  estimate is equivalent to the posterior mean when assuming a uniform prior distribution, and improves upon the poor performance [22] of the usual estimate  = *D*_*i*_/*F*_*i *_when *D*_*i *_= 0. If the actual p-value cutoff *p*_0 _is outside the *c*-level confidence interval for *p*_*i *_then marker *i *will not be included for resample allocation in the next round. The confidence interval is approximated by a normal distribution with mean  and standard deviation . We use *c *= 0.01,0.05, 0.1, 0.3, 0.5 in our simulation.

5. For our algorithm, *B*_0 _= *K *= 10, *B *= 100.

## Results and Discussion

### Experimental Validation

We applied our algorithm to two different datasets to check how efficient it is compared with the conventional uniformly-allocated re-sampling. The first dataset is a publicly available microarray dataset to detect genes differentially expressed across two conditions. The second, much larger dataset, is a publicly-available genome-wide assocation study on Parkinson's disease [18].

#### Application to a breast cancer microarray dataset

**Figure 2:**
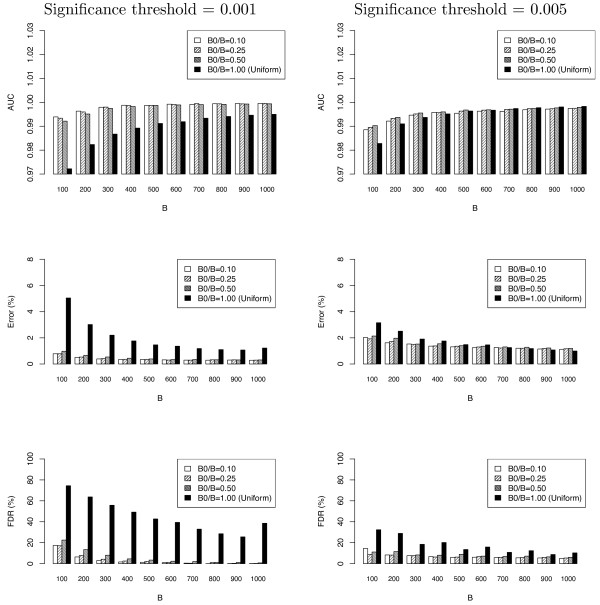
**Sample figure title**. Area under ROC curve (AUC), Error (defined as (FN+FP)/(P+N)), and false discovery rate (FDR) of the uniform (*B/B*_0 _= 1) and differential allocation algorithms using the Hedenfalk et al. gene expression dataset. Left: p-value cutoff = 0.001; right: p-value cutoff = 0.005.

Our algorithm was first applied to the microarray dataset presented in [17]. The details of preprocessing and application of the algorithm to this data are presented in the Methods Section. The results are in Figure 2. We observe that in the microarray dataset, the differential allocation algorithm (*B*_0_/*B *< 1) outperforms the uniform allocation algorithm (*B*_0_/*B *= 1) substantially, though the gap becomes smaller when *B *increases. We also measured the areas under ROC curve to eliminate the effect of selecting a particular threshold of significance, and observed the same trends (data not shown). In both datasets, the choice of burn-in proportion *B*_0_/*B *for the differential allocation algorithm did not seem to affect the performance of the algorithm.

#### Application to a Parkinson disease genome-wide association study dataset

The results from the previous section suggest our algorithm has the best improvement over the uniform allocation when the number of possible resamples is relatively small. In this section, we test our algorithm on a publicly-available genome-wide association study where the number of possible resamples is relatively large. Typical datasets in genome-wide association (GWA) studies may consist of several thousand case and control subjects each, using single nucleotide polymorphism (SNP) genotyping arrays that can genotype up to 10^6 ^SNPs. The most common goal of a genome-wide association study is to find SNP(s) that are highly correlated with the case/control status. One simple way to test the association is to run chi-square tests on the two-way 3 × 2 contingency table between the genotype of each SNP (zero, one, or two copies of the minor allele) and the case-control status [23]. Existence of such SNPs suggests nearby genomic regions may carry significant genes, regulatory motifs, or other DNA sequences that may affect the disease risk.

This setting is an important test of computationally efficient resampling-based procedures for several important reasons. First, the high number of SNPs being tested implies a very stringent p-value threshold if we take the issue of multiple testing into consideration: setting p-value cutoff at 10^-5 ^or lower is typical, so any resampling-based p-value computation for each SNP requires at least 10^5 ^resamples if uniform allocation is used. Second, the high number of subjects means evaluating the test statistic for each resample is more costly. Finally, although we focus on simple chi-square tests as a proof of concept for our procedure, even more complex and computational demanding tests that may involve interactions between multiple SNPs and pedigree information relating subjects are being actively developed and applied to improve the sensitivity of GWA studies. As a example, it is common to consider the maximum p-value between multiple tests, such as an allelic test and a genotypic test, in a GWA analysis. These tests may employ statistics that are computationally expensive, and p-values have to be evaluated using resampling if exact p-value formulas are not available.

**Figure 3:**
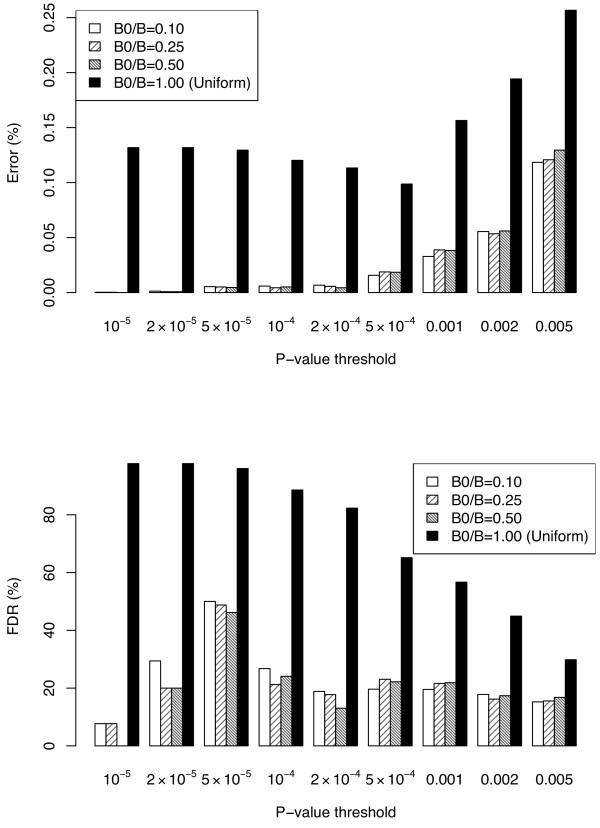
**Sample figure title**. Error (defined as (FN+FP)/(P+N)) and false discovery rate of the uniform (*B/B*_0 _= 1) and differential allocation algorithms using the public Parkinson Disease genome-wide association study dataset.

As an illustration of our procedure in this difficult setting, we applied our algorithm to a public Parkinson genome-wide association study dataset [18]. Refer to the Methods Section on details of the dataset and the application of our algorithm. The results are summarized in Figure 3. Notice that our procedure has excellent accuracy and false discovery rate: at most 20% except when the p-value cutoff is 5 × 10^-5^. Moreover, the proportion of "burn-in" permutation resamples has little effect on the accuracy of the differential allocation algorithm, probably because the enormous number of total SNPs implies there are always enough resamples from nonsignificant SNPs to be reallocated when needed. Nonuniform allocation always outperforms uniform allocation by a substantial amount. Since *B *= 1000, for p-value thresholds lower than 10^-4 ^only SNPs with 0 as their estimated p-values can pass the threshold under the uniform allocation. The uniform allocation algorithm has much higher error and false discovery rate because p-value estimation for significant and borderline SNPs is less accurate, and the situation is not improved even when the p-value threshold increases to 10^-3^.

#### Simulation comparison to shortcut method

In addition to demonstrating increased efficiency over a uniform allocation scheme, we also evaluate our method against the shortcut method, which is also described in our Methods section. We use *N *= 300, 000 markers, typical for genome-wide association studies. We generate the "actual" 300,000 p-values following a uniform distribution since we know that the p-values of all (but a few) markers should be uniformly distributed in a well-designed genome-wide association study where no confounding factors such as population stratification exist. We evaluate our performance relative to the shortcut method using simulated p-values directly. See the Methods section on details of the simulation.

**Figure 4:**
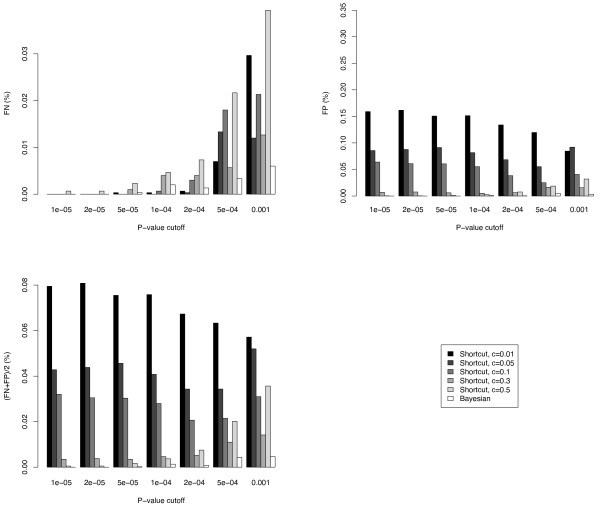
**Sample figure title**. FN, FP, and Average Error (defined as (FN+FP)/2) of the shortcut and our differential allocation algorithm (bayesian) in our simulation study. The value *c *in the legend is the level of confidence interval used in the shortcut method; see text for more details.

Please see Figure 4 for the results of the simulation. As can be seen, our method consistently outperforms the shortcut method. Note that as we increase the p-value cutoff, the FN rate increases and the FP rate decreases for the shortcut method. Moreover, when the value of confidence level *c *increases in the shortcut method, the FP rate decreases but the FN rate varies in a more complex pattern affected by both the confidence level *c *and *p*_0_. Small values of *c *in the shortcut method has good FN rate in general (and outperforms the bayesian method for *p*_0 _= 10^-4 ^and 2 × 10^-4^, but the FP rate is too high. On the other hand, a large setting of *c *has good FP rate and bad FN rate. These observations hint that a symmetric test for both FP and FN in the shortcut method may be suboptimal (using the same value of *c*, level of confidence interval for both FP and FN scenarios) and an asymmetric approach such as our algorithm is preferred. Another contributing factor is that our approach is more global in the sense of allocating resamples proportional to the risks across all markers as opposed to the shortcut approach that treats each marker independently of the progression of other markers.

#### Running time

We explored the overhead associated with our differential allocation approach and found it to be negligible on a modern computer. We computed the running time of the shortcut method and our differential allocation algorithm for 5 repetitions of our simulation involving 300,000 SNPs on a dual-quad-core Xeon linux server using R (64-bit version 2.8.1; our implementation is single-threaded and no parallelization is involved). Since the permutation tests in this simulation are generated by random p-values, the running time is almost entirely a function of the overhead of the allocation algorithms, not the individual statistical tests. The average running time of shortcut method in this situation was about 3.5 minutes, and the average running time of our algorithm was 1 minute, suggesting that neither method has substantial overhead. Certainly in a situation with more complex individual statistical tests, the running time of either approach will be dominated by the unavoidable calculations of each test statistic.

## Conclusion

In this paper we presented a new approach to more efficiently assign resamples (such as bootstrap samples or permutations) within a nonparametric multiple testing framework. We formulated a Bayesian-inspired approach to this problem, and devised an algorithm that adapts the assignment of resamples iteratively with negligible space and running time overhead. In two experimental studies, a breast cancer microarray dataset and a genome wide association study dataset for Parkinson's disease, we demonstrated that our differential allocation procedure is substantially more accurate compared to the traditional uniform resample allocation. In a simulation study we showed our algorithm outperforms the simpler shortcut method under various settings. It is worth emphasizing that our methodology is not ideally suited for the accurate estimation of all p-values, especially p-values far from the significance threshold (in either direction). Rather, our methodology focusses on the accuracy of significance decisions by ensuring that p-values near the decision threshold are most accurately estimated.

The idea of using a non-uniform search among a large number of tests is quite common in other multiple testing situations. An example is efficient variable selection in regression models where the number of covariates is very large. Similar applications can also be found elsewhere: in finance, [24] used a stepwise regression procedure to predict bankruptcy, where significant predictors are added (from a large pool of possible predictors) sequentially using a procedure where there is differential allocation for the threshold of significance. Techniques such as this are different from our situation since we are taking a non-parametric approach to a simpler testing situation, but we still share the similar idea that one can gain power by differentially allocating resources towards the tests that are most likely to be significant. When individual tests are simple to compute, e.g., Fisher's exact test on small contingency tables when the p-value can be computed exactly, the gain by our algorithm or other differential allocation methods is limited. However, a differential allocation approach is much more important when more computationally intensive tests are used, such as in Gene Set Enrichment Analysis [25], or family-based association tests in genome-wide association studies [26].

## Authors' contributions

SJ and LW designed this study and developed the new algorithm. LW coded the new algorithm and the shortcut method. SS and LW ran the experiments. All three authors wrote and approved the manuscript.
